# Supplementary material for: Renewable energy production will exacerbate mining threats to biodiversity
Source: Nat Commun. 2020 Sep 1;11:4174. doi: 10.1038/s41467-020-17928-5 (PMC7463236; doi:10.1038/s41467-020-17928-5)
Supplement: Supplementary file 1 — Supplementary Information [file 41467_2020_17928_MOESM1_ESM.pdf]

SUPPLEMENTARY MATERIALS

Sonter et al.

Renewable energy production will exacerbate mining threats to biodiversity

FILE CONTAINS:

- Supplementary Figures 1–3
- Supplementary Tables 1–4
- References cited in Supplementary Materials

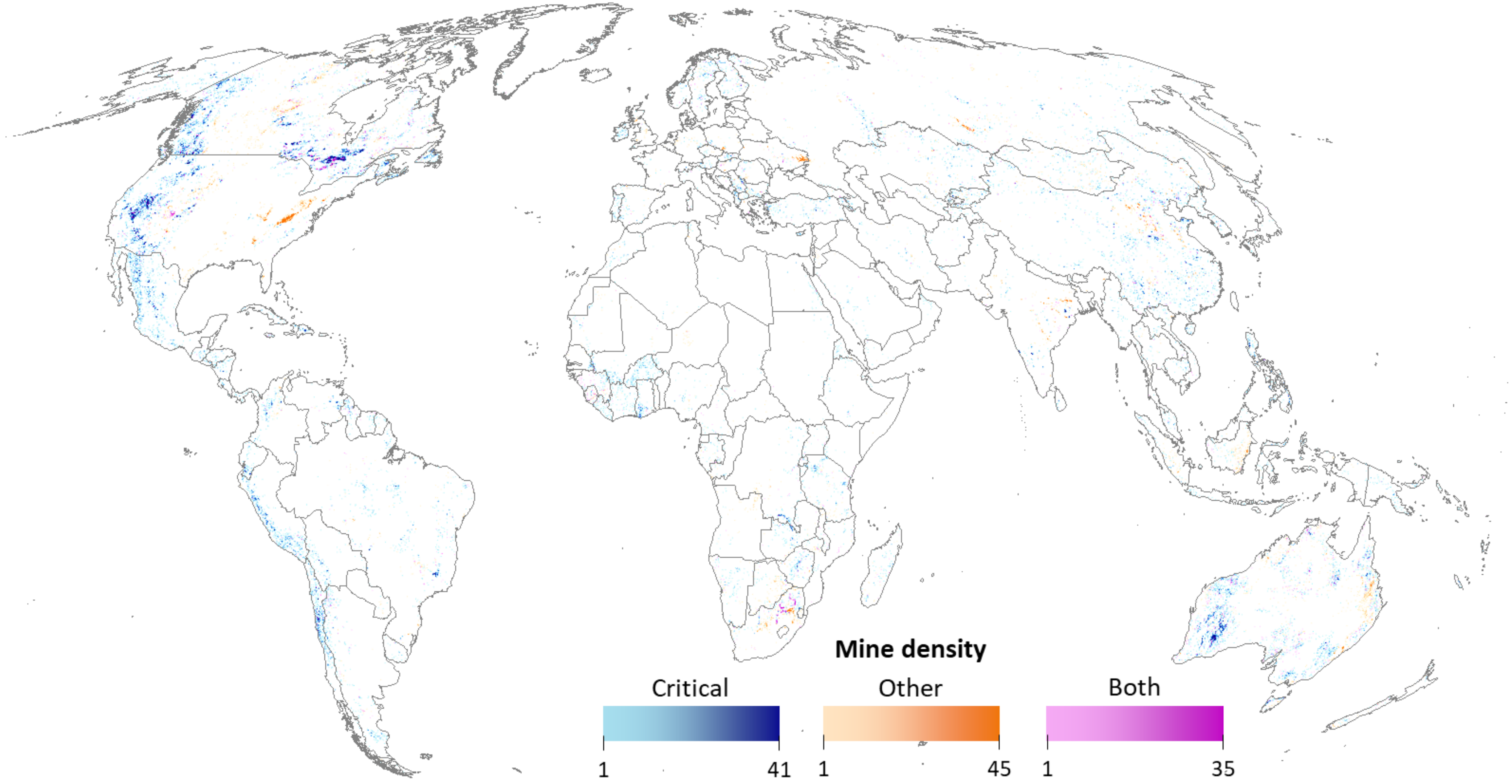

Supplementary Figure 1: Global mining areas and their density. Mining areas were mapped using a 10-cell radius around 62,381 pre-operational, operational and closed mining properties. Mining areas with properties targeting materials critical for renewable energy technology and infrastructure are shown in blue, areas with properties targeting other materials are shown in orange, and those targeting both commodity types are shown in pink. Colour shading (light to dark) indicates the density of mining areas – i.e. the number of mining properties within a 10-cell radius of each 1 km cell.

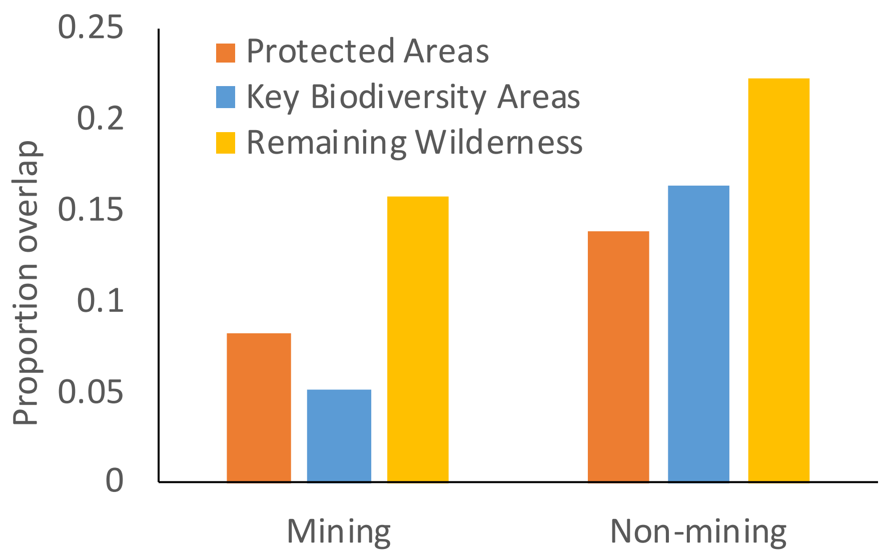

Supplementary Figure 2: Overlap between mining and biodiversity conservation. Bars depict the areal proportion of mining and non-mining areas that overlap with currently Protected Areas and conservation priorities (Key Biodiversity Areas and Remaining Wilderness). Mining areas were mapped using a 10-cell radius around known mining properties; non-mining areas include the terrestrial land surface further than 50-cells from a mining property and excluding Antarctica. Mining areas overlapped with Protected Areas and Remaining Wilderness significantly less often than non-mining regions overlapped with Protected Areas and conservation priorities ($p<0.05$; Supplementary Data 1), although differences for Key Biodiversity Areas were marginal ($p=0.01$; Supplementary Data 1). Note: proportions were calculated from full dataset, whereas statistics were calculated using the data sampled at 100-cell intervals.

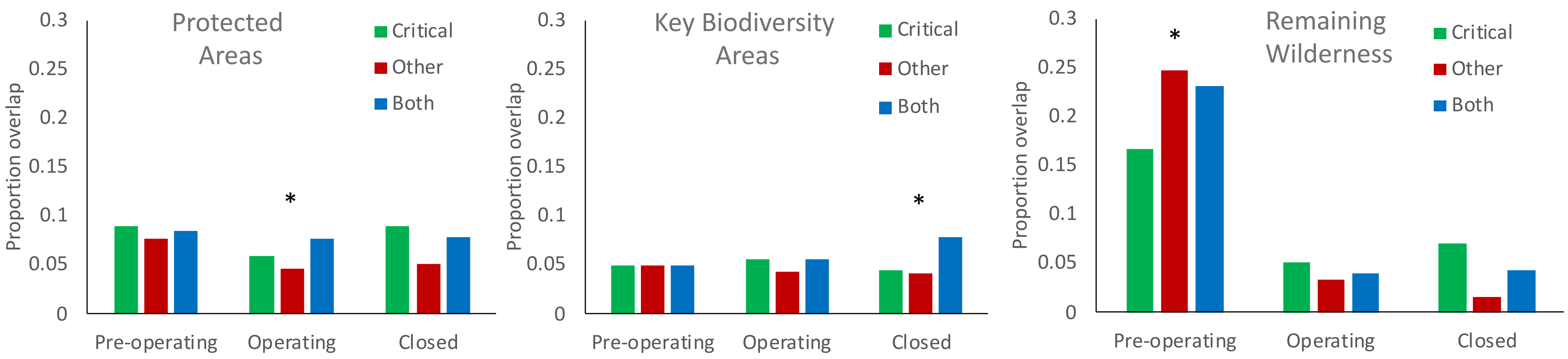

Supplementary Figure 3: Overlap between critical mining areas and biodiversity conservation. Bars depict the areal proportion of critical, other and both mining areas that overlap with currently Protected Areas and conservation priorities (Key Biodiversity Areas and Remaining Wilderness). Mining areas were mapped using a 10-cell radius around known mining properties, separated into those that are listed as being in pre-operational, operational and closed phases. See Supplementary Data 1 for test statistics. Stars denote significance differences among columns (critical, other, both) within a mining phase (i.e. pre-operational, operational, closed; *$p<0.05$). Note: proportions shown on Figure were calculated from full dataset, whereas statistics were calculated using the data sampled at 100-cell intervals.

Supplementary Table 1: Number of mining properties targeting materials required for renewable energy technology and infrastructure vs. other non-renewable commodities.

Critical for renewables	C1	C2	C3	C4	C5	C6	Total
Gold	13093	2469	779	638	374	130	**17483**
Copper	4404	2380	1077	435	281	84	**8661**
Silver	1091	3248	1622	776	337	82	**7156**
Zinc	1097	1104	964	643	255	67	**4130**
Lead	268	1060	903	648	344	55	**3278**
Iron Ore	1820	171	139	82	46	20	**2278**
Nickel	1170	346	128	110	111	52	**1917**
Molybdenum	295	543	302	170	69	52	**1431**
Cobalt	114	401	259	97	63	78	**1012**
Platinum	325	163	293	148	60	21	**1010**
Palladium	24	305	135	279	131	59	**933**
Lanthanides	324	82	46	25	29	11	**517**
Lithium	392	38	14	15	6	13	**478**
Tin	218	82	45	26	16	17	**404**
Tungsten	129	109	70	37	32	23	**400**
Manganese	195	61	32	22	12	10	**332**
Graphite	299	4	13	2	6	2	**326**
Vanadium	66	117	41	31	16	10	**281**
Bauxite	233	2	3		2		**240**
Tantalum	70	83	51	15	6	5	**230**
Chromite	148	13	23	7	9	6	**206**
Antimony	51	50	31	16	13	14	**175**
Titanium	19	75	44	10	19	7	**174**
Niobium	33	63	38	21	11	3	**169**
Zircon	4	14	121	19	4	2	**164**
Yttrium	2	19	17	14	9	1	**62**
Scandium	7	8	9	6	3	4	**37**
Chromium	1	4	4	3	6	3	**21**
Alumina	1	2	1		1		**5**
Aluminum				1			**1**
Platinum Group Metals					1		**1**
**Total**	**25594**	**13012**	**7191**	**4294**	**2266**	**829**	**53186**

Not critical	C1	C2	C3	C4	C5	C6	Total
Coal	5172						**5172**
U3O8	1668	133	104	46	31	22	**2004**
Diamonds	1439	48	7	8	3	8	**1513**
Phosphate	256	35	10	11	3	5	**320**
Potash	202	48	5	2	2		**259**
Ilmenite	129	23	12	8	5	2	**179**
Rutile	13	111	20	6			**150**
Borates		5	2	1			**8**
**Total**	**9195**	**412**	**176**	**197**	**58**	**41**	**10079**

"C1" indicates primary commodities, "C2" indicates secondary commodities, etc. Note some mines produce commodities listed as renewable and non-renewable in this table. Commodity list from World Bank report[1]; mining property data from S&P database[2]

Supplementary Table 2: Summary statistics for mining areas. Summary statistics (minimum, maximum, mean and standard deviations) were calculated from the full dataset, rather than dataset sampled at 100-cell intervals.

		**Mining areas defined using 50-cell radius (see Figure 1)**					**Mining areas defined using 10-cell radius (see Supplementary Figure 1)**				
**Status**	**Commodity**	**Area (km^2)**	**Mining density**				**Area (km^2)**	**Mining density**			
			**Min**	**Max**	**Mean**	**St dev**		**Min**	**Max**	**Mean**	**St dev**
**All**	**All**	**49872901**	**1**	**235**	**4.89**	**9.03**	**6669253**	**1**	**45**	**1.63**	**1.59**
	Critical	25356935	1	138	3.57	5.44	1481009	1	45	1.62	1.78
	Other	8920656	1	235	3.79	10.69	4457797	1	41	1.56	1.49
	Both	15595310	1	228	8.95	13.28	730108	1	35	2.12	1.99
**Pre-operational**	**All**	**42143922**	**1**	**199**	**4.36**	**8.19**	**5186911**	**1**	**34**	**1.47**	**1.24**
	Critical	23601124	1	107	3.16	4.73	3695997	1	34	1.47	1.26
	Other	6802731	1	39	2.74	3.36	944373	1	17	1.20	0.58
	Both	11740067	1	199	8.16	12.49	546541	1	29	1.93	1.73
**Operational**	**All**	**19650402**	**1**	**107**	**2.14**	**2.58**	**1544896**	**1**	**23**	**1.44**	**1.16**
	Critical	11157490	1	51	1.96	2.26	867003	1	20	1.25	0.78
	Other	5341214	1	107	3.46	6.56	555662	1	23	1.69	1.53
	Both	3151698	1	89	4.35	5.38	122231	1	15	1.62	1.20
**Closed**	**All**	**6784468**	**1**	**75**	**1.82**	**3.25**	**409019**	**1**	**20**	**1.25**	**0.84**
	Critical	4339981	1	15	1.37	0.89	239870	1	5	1.10	0.35
	Other	1818984	1	75	2.73	5.85	145243	1	20	1.49	1.27
	Both	625503	1	21	2.32	2.35	23906	1	9	1.31	0.77

Supplementary Table 3: Development stage for mining properties, reclassified into three statuses (pre-operational, operational and closed) for analysis in this study.

**Pre-operational**		**Operational**		**Closed**	
Grass roots	5140	Preproduction	280	Closed	1634
Exploration	7467	Construction planned	95		
Target outline	4645	Construction started	178		
Reserves development	4005	Commissioning	30		
Advanced exploration	1115	Operating	5415		
Prefeasibility/scoping	914	Satellite	535		
Feasibility	676	Expansion	312		
Feasibility started	203	Limited production	234		
Feasibility complete	200	Residual production	26		
Total	***24365***		***7105***		***1634***

Supplementary Table 4: Spatial correlation in mining density, obtained from spatial correlograms constructed in GeoDa[3] using 50,000 randomly selected cells and 1 million sample pairs.

Distance band (km)	Mining areas created using a 50-cell radius			Mining areas created using a 10-cell radius		
	Critical	Other	Both	Critical	Other	Both
0–50	0.164	0.009	0.514	0.376	-0.003	0.033
50–100	0.095	0.017	0.147	0.060	-0.002	-0.002
100–150	0.074	0.002	0.103	0.009	-0.003	0.026
150–200	0.043	0.001	0.116	0.071	-0.001	-0.006
200–250	0.024	0.005	0.064	-0.008	-0.003	0.006
250–300	0.041	-0.001	0.058	0.004	-0.002	-0.002
300–350	0.021	-0.001	0.023	0.005	-0.002	-0.004
350–400	0.009	0.002	0.012	-0.006	-0.001	-0.004
400–450	0.024	0.009	0.006	0.023	-0.003	-0.003
450–500	0.016	-0.000	0.030	0.019	0.002	-0.002
500–550	0.008	0.009	0.027	0.013	0.006	-0.003
550–600	-0.007	-0.001	0.022	0.005	-0.003	-0.003
600–650	-0.003	0.004	0.018	0.008	0.001	-0.001
650–700	-0.001	0.001	0.017	0.006	0.073	-0.001
700–750	0.017	0.004	0.004	0.001	0.005	-0.001
750–800	0.056	-0.000	0.021	0.005	-0.021	-0.001
800–850	0.056	0.001	0.021	0.021	-0.002	-0.001
850–900	0.036	-0.002	0.051	0.015	0.000	0.005
900–950	0.036	0.001	0.024	0.015	0.028	0.000
950–1000	0.012	-0.002	0.012	0.014	0.001	-0.002

References cited in Supplementary Materials

1 IEA. Energy Technology Perspectives 2015: Mobilising Innovation to Accelerate Climate Action. (International Energy Agency, Paris, 2015).
2 S&P. S&P Global Market Intelligence. (New York, 2018).
3 Global Spatial Autocorrlation (1): Moran Scatter Plot and Spatial Correlogram (2018).
