# Supplementary material for: Renewable energy production will exacerbate mining threats to biodiversity
Source: Nat Commun. 2020 Sep 1;11:4174. doi: 10.1038/s41467-020-17928-5 (PMC7463236; doi:10.1038/s41467-020-17928-5)
Supplement: Supplementary file 3 — Description of Additional Supplementary Files [file 41467_2020_17928_MOESM3_ESM.pdf]

Description of Additional Supplementary Files

File Name: Supplementary Data 1
Description: Chi-squared test statistics comparing spatial overlap with protected areas and conservation priorities. Comparisons made between mining and non-mining areas (columns D and E) and between critical vs. other mining areas (columns H and I). Differences are also shown between mining areas defined using a 50-cell radius around mining properties and those defined using a 10-cell radius, and when sampling the mining regions at 100 km and 300 km interval.

File Name: Supplementary Data 2
Description: Summary statistics and Kolmogorov-Smirnov (D) test statistics comparing mining density between mining areas overlapping with conservation and mining areas not overlapping with conservation. Differences are also shown between mining areas defined using a 50-cell radius around mining properties and those defined using a 10-cell radius, and when sampling the mining regions at 100 km and 300 km intervals.

File Name: Supplementary Data 3
Description: Summary statistics and Kolmogorov-Smirnov (D) test statistics comparing mining density between critical mining areas overlapping with conservation and other mining areas overlapping with conservation. Differences are also shown between mining areas defined using a 50-cell radius around mining properties and those defined using a 10-cell radius, and when sampling the mining regions at 100 km and 300 km intervals.
