# Supplementary material for: Renewable energy production will exacerbate mining threats to biodiversity
Source: Nat Commun. 2020 Sep 1;11:4174. doi: 10.1038/s41467-020-17928-5 (PMC7463236; doi:10.1038/s41467-020-17928-5)
Supplement: Supplementary file 7 — Reporting Summary [file 41467_2020_17928_MOESM7_ESM.pdf]

nature research

Corresponding author(s): DBPR NCOMMS-19-539405A

Last updated by author(s): Jul 7, 2020

Reporting Summary

Nature Research wishes to improve the reproducibility of the work that we publish. This form provides structure for consistency and transparency in reporting. For further information on Nature Research policies, see our Editorial Policies and the Editorial Policy Checklist.

Statistics

For all statistical analyses, confirm that the following items are present in the figure legend, table legend, main text, or Methods section.

n/a	Confirmed	
☐	☒	The exact sample size (n) for each experimental group/condition, given as a discrete number and unit of measurement
☐	☒	A statement on whether measurements were taken from distinct samples or whether the same sample was measured repeatedly
☐	☒	The statistical test(s) used AND whether they are one- or two-sided<br>*Only common tests should be described solely by name; describe more complex techniques in the Methods section.*
☒	☐	A description of all covariates tested
☐	☒	A description of any assumptions or corrections, such as tests of normality and adjustment for multiple comparisons
☐	☒	A full description of the statistical parameters including central tendency (e.g. means) or other basic estimates (e.g. regression coefficient) AND variation (e.g. standard deviation) or associated estimates of uncertainty (e.g. confidence intervals)
☒	☐	For null hypothesis testing, the test statistic (e.g. F, t, r) with confidence intervals, effect sizes, degrees of freedom and P value noted<br>*Give P values as exact values whenever suitable.*
☒	☐	For Bayesian analysis, information on the choice of priors and Markov chain Monte Carlo settings
☒	☐	For hierarchical and complex designs, identification of the appropriate level for tests and full reporting of outcomes
☒	☐	Estimates of effect sizes (e.g. Cohen's d, Pearson's r), indicating how they were calculated

Our web collection on statistics for biologists contains articles on many of the points above.

Software and code

Policy information about availability of computer code

Data collection	No software was used in the collection of data.
Data analysis	We used ArcGIS (version 10.7.1) to create maps of mining regions, QGIS (version 3.12) to sample mining regions, GeoDa (version 1.14.0) to analyse spatial autocorrelation, and R (version 3.4) to perform statistical tests.

For manuscripts utilizing custom algorithms or software that are central to the research but not yet described in published literature, software must be made available to editors and reviewers. We strongly encourage code deposition in a community repository (e.g. GitHub). See the Nature Research guidelines for submitting code & software for further information.

Data

Policy information about availability of data

All manuscripts must include a data availability statement. This statement should provide the following information, where applicable:

- Accession codes, unique identifiers, or web links for publicly available datasets
- A list of figures that have associated raw data
- A description of any restrictions on data availability

Spatial data on Protected Areas and Conservation Priorities are available from refs 21, 24 and 25. The mining properties database (ref 31) is not freely available, but the global mining areas maps that we produced in this study (1km2 resolution, using 50-cell radius, per commodity type (critical, other and both; Figure 1) can be downloaded as tiff files from 10.6084/m9.figshare.12630092. All other combinations of mining areas (i.e. by status [pre-operational, operational and closed] and when using 10-cell radius; Supplementary Figure 1) can be obtained by contacting the corresponding author.

Field-specific reporting

Please select the one below that is the best fit for your research. If you are not sure, read the appropriate sections before making your selection.

☐ Life sciences ☐ Behavioural & social sciences ☒ Ecological, evolutionary & environmental sciences

For a reference copy of the document with all sections, see nature.com/documents/nr-reporting-summary-flat.pdf

Ecological, evolutionary & environmental sciences study design

All studies must disclose on these points even when the disclosure is negative.

Study description	We mapped areas influenced by mining and quantified their overlap with important biodiversity conservation sites. We compared proportional overlap between mining areas targeting materials critical for renewable energy production and mining areas targeting other materials.
Research sample	We examined areas influenced by mining across the Earth's land surface. We mapped these areas as 1 km grid cells occurring within 50 km of a mining property. Data on 62,381 mining properties were obtained from S&P Global Market Intelligence (ref 28).
Sampling strategy	We sampled data at 100 km intervals to remove potential issues with large sample sizes and the spatial autocorrelation created in our dataset by mapping mining areas using a 50-cell radius around each mining property. Sampling at 100-cell intervals also removed most other sources of autocorrelation (Supplementary Table 4). To test the robustness of this sampling strategy, we repeated our analyses when sampling at 300 km intervals (Supplementary Data 1 - 3).
Data collection	Mining properties were obtained by LJS from S&P Global Market Intelligence (ref 31). Current extents of protected areas, key biodiversity areas and remaining wilderness were obtained from references 24, 21 and 25 respectively.
Timing and spatial scale	Data was analyzed at the global scale.
Data exclusions	No data were excluded
Reproducibility	NA
Randomization	NA - we did not perform experiments.
Blinding	NA

Did the study involve field work? ☐ Yes ☒ No

Reporting for specific materials, systems and methods

We require information from authors about some types of materials, experimental systems and methods used in many studies. Here, indicate whether each material, system or method listed is relevant to your study. If you are not sure if a list item applies to your research, read the appropriate section before selecting a response.

Materials & experimental systems

n/a	Involved in the study	
☒	☐	Antibodies
☒	☐	Eukaryotic cell lines
☒	☐	Palaeontology and archaeology
☒	☐	Animals and other organisms
☒	☐	Human research participants
☒	☐	Clinical data
☒	☐	Dual use research of concern

Methods

n/a	Involved in the study	
☒	☐	ChIP-seq
☒	☐	Flow cytometry
☒	☐	MRI-based neuroimaging
